# Multi temporal multispectral UAV remote sensing allows for yield assessment across European wheat varieties already before flowering

**DOI:** 10.3389/fpls.2023.1214931

**Published:** 2024-01-03

**Authors:** Moritz Paul Camenzind, Kang Yu

**Affiliations:** ^1^ Precision Agriculture Lab, School of Life Sciences, Technical University of Munich, Freising, Germany; ^2^ World Agricultural Systems Center (Hans Eisenmann-Forum for Agricultural Sciences – HEF), Technical University of Munich, Freising, Germany

**Keywords:** wheat variety testing, yield prediction, UAV remote sensing, image texture features, machine learning, phenology

## Abstract

High throughput field phenotyping techniques employing multispectral cameras allow extracting a variety of variables and features to predict yield and yield related traits, but little is known about which types of multispectral features are optimal to forecast yield potential in the early growth phase. In this study, we aim to identify multispectral features that are able to accurately predict yield and aid in variety classification at different growth stages throughout the season. Furthermore, we hypothesize that texture features (TFs) are more suitable for variety classification than for yield prediction. Throughout 2021 and 2022, a trial involving 19 and 18 European wheat varieties, respectively, was conducted. Multispectral images, encompassing visible, Red-edge, and near-infrared (NIR) bands, were captured at 19 and 22 time points from tillering to harvest using an unmanned aerial vehicle (UAV) in the first and second year of trial. Subsequently, orthomosaic images were generated, and various features were extracted, including single-band reflectances, vegetation indices (VI), and TFs derived from a gray level correlation matrix (GLCM). The performance of these features in predicting yield and classifying varieties at different growth stages was assessed using random forest models. Measurements during the flowering stage demonstrated superior performance for most features. Specifically, Red reflectance achieved a root mean square error (RMSE) of 52.4 g m^-2^ in the first year and 64.4 g m^-2^ in the second year. The NDRE VI yielded the most accurate predictions with an RMSE of 49.1 g m^-2^ and 60.6 g m^-2^, respectively. Moreover, TFs such as CONTRAST and DISSIMILARITY displayed the best performance in predicting yield, with RMSE values of 55.5 g m^-2^ and 66.3 g m^-2^ across the two years of trial. Combining data from different dates enhanced yield prediction and stabilized predictions across dates. TFs exhibited high accuracy in classifying low and high-yielding varieties. The CORRELATION feature achieved an accuracy of 88% in the first year, while the HOMOGENEITY feature reached 92% accuracy in the second year. This study confirms the hypothesis that TFs are more suitable for variety classification than for yield prediction. The results underscore the potential of TFs derived from multispectral images in early yield prediction and varietal classification, offering insights for HTP and precision agriculture alike.

## 1 Introduction

Yield improvements are currently estimated to average less than 1% annually in Europe and are even decreasing in some European countries (Ray et al., 2013). One of the reasons for this stagnation are low breeding gains which are estimated to be only 0.45% (Cormier et al., 2013) per year. Grain yield is the product of the number of grains per area and the weight of a single grain, which are both controlled by a variety of genes. New molecular tools have emerged to advance breeding for such quantitative traits but their potential is still not exploited, partly due to our ability to phenotype (Araus and Cairns, 2014). Traditional methods for phenotyping of yield and yield related traits often require manual labor and are thus slow, expensive and subjective. Faster, cheaper and standardizable alternatives have emerged quickly in recent years and are referred to as high-throughput phenotyping (HTP) (Cabrera-Bosquet et al., 2012; Hund et al., 2019; Watt et al., 2020).

HTP employs a variety of advanced technologies such as digital imaging, remote sensing and artificial intelligence but to assess grain yield directly remains infeasible under field conditions. Major advances have been achieved in counting the number of spikes (David et al., 2020; David et al., 2021) and first attempts have been made to count the number of grains per spike (Xu et al., 2023). To our knowledge, grain weight has not been directly assessed under field conditions using remote sensing. Although these techniques are promising, they are based on computer vision and require images that show a high level of detail, resulting in a low throughput of the technology (Eskandari et al., 2020). To overcome this limitation, yield assessment often focuses on the estimation of secondary traits that are related to yield formation (Li et al., 2019a).

To identify suitable secondary traits, yield formation has to be well understood. With an average precipitation between 2010 and 2020 of 741 mm (Climate Data Center of the German Weather Service), the agricultural systems in the Freising District, Bavaria, Germany can be classified as radiation limited (Patrignani et al., 2014), although lack of precipitation and high temperatures can lead to yield losses in this region as well (Heil et al., 2023). Therefore, yield formation in this region can very broadly be described as a function of the incident radiation per day during the growing season, the intercepted radiation over the canopy life cycle, the green leaf duration, the radiation use efficiency as well as the harvest index (Araus et al., 2008). This indicates that a single time point may not be sufficient for an accurate yield assessment. Furthermore, yield formation is influenced by an interplay of sources and sinks. The sinks can be seen as the potential yield and sources as the actual supply of assimilates (Fischer, 2011). This interplay starts with the transition of the plants from the vegetative to the reproductive stage and continues during anthesis until the grain filling stage (Slafer and Rawson, 1994). Still, some stages are more critical for yield formation than others are. Fischer (1985) found that a relatively short period before flowering is critical for yield formation due to the source driven survival of floret primordia at the stem elongation stage (González et al., 2005) and is linked to the spike biomass (Slafer et al., 1996). Breeders however are interested in the yield potential as early as possible in the growing season in order to be able to focus their phenotyping efforts on well performing genotypes (Garriga et al., 2017). At the germination stage, the maximum number of plants and at the tillering stage, the maximum number of tillers is being formed which are all linked to the final number of grains harvested. However, the tillering potential is highly dependent on the environment and under high yielding environments no differences in yield were found between varieties with a low and such with a high tillering potential (Bastos et al., 2020). Furthermore, the sinks at these early stages are microscopically small and hidden in the developing stems, making their detection impossible by remote sensing technologies. Therefore, predicting yield at the tillering stage is difficult.

Secondary traits related to the sources such as leaf area index (LAI) (Bukowiecki et al., 2020), chlorophyll content (Pan et al., 2023) and finally biomass (Yue et al., 2019) have been phenotyped using a variety of techniques. Primary traits such as grain yield and quality have been assessed by estimating the mentioned secondary traits during the growth season (Duan et al., 2017; Hassan et al., 2019; Vatter et al., 2022). A variety of sensors have been employed such as RGB cameras (Fernandez-Gallego et al., 2019), multispectral cameras (Prey et al., 2022), hyperspectral sensors (Bowman et al., 2015), thermal cameras (Elsayed et al., 2017) and active sensors such as Lidar (Li et al., 2022) to mention a few. Among these technologies, multispectral cameras offer a high work efficiency for a relatively low cost. Along with the reflectance, multispectral cameras are imaging sensors and therefore have the advantage of capturing the structure or texture of an object. As a result, they allow for extracting a unique variety of features to assess yield in wheat. Generally, these features can be grouped into three categories. First, single-band reflectance in specific wavelengths can be directly extracted from multispectral data. Vatter et al. (2022) fed single band reflectances to a neural network and predicted durum wheat quality and yield before the harvest. Second, the reflectance of single bands can be combined to calculate vegetation indices (VIs), which are often more sensitive to specific traits and less affected by environmental conditions during measurement (Tucker, 1979). This approach has been used by several studies for yield prediction (Duan et al., 2017; Hassan et al., 2019; Prey et al., 2022). However, single-band reflectance and VIs may suffer from saturation, particularly when the canopies are closed (Rischbeck et al., 2016). Third, texture features (TFs) can be extracted to describe the distribution of pixels within a region of interest (ROI). TFs were originally designed for image classification (Haralick et al., 1973) and have since been used for classification of forest stands (Coburn and Roberts, 2004), wheat phenology (Zhou et al., 2023) and wheat seeds (Khojastehnazhand and Roostaei, 2022). Therefore, they might also be beneficial when identifying elite wheat varieties directly as suggested by Garriga et al. (2017). Several studies employed TFs for yield prediction in explorative studies and found that they often perform less effectively than single-band reflectance or VIs (Li et al., 2019b; Yue et al., 2019; Zhang et al., 2021) but can improve the prediction of leaf area and biomass when combined with VIs (Yue et al., 2019; Zhang et al., 2021).

Accurately predicting yield or identifying elite wheat varieties using multispectral reflectance further requires careful consideration of the phenological stage of the canopy. Late stages such as the anthesis stage and the grain filling stage are often identified as the most suitable stages for yield prediction in wheat when using VIs (Bowman et al., 2015; Duan et al., 2017; Hassan et al., 2019). Earlier stages such the tillering stage are generally and naturally performing worse (Prey et al., 2022). Still, Walsh et al. (2022) successfully predicted yield at the tillering stage and Marti et al. (2007) at the stem elongation stage, both using the normalized difference vegetation index (NDVI). To date, most studies focus on only one measurement date during a given phenological stage or test only one feature or feature class. A more detailed study is therefore needed to better understand the interaction of phenology stages and features for yield prediction. Particularly, phenology showed to have a big influence on the relationship between biomass and TFs (Li et al., 2019c). However, the performance of TFs at different phenological stages has been reported in a few studies only (Zhang et al., 2021). Multispectral cameras mounted on unmanned aerial vehicles (UAVs) further enable breeders and researchers to assess the aforementioned spectral and TFs at a high temporal frequency and precision. Within a proper time-window, using a time series for yield prediction allows for the extraction of dynamic canopy traits that could potentially be useful for yield prediction. For instance, Pinter et al. (1981) suggested summing measurement dates after heading to improve yield prediction in wheat and barley whereas Raun et al. (2001) suggested taking two spectral measurements after dormancy. Prey et al. (2022) showed that models containing data from multiple dates could improve yield predictions and compensate if data could not be collected on the optimal date due to practical reasons and phenological shifts between years.

Collectively, despite these successes, little is known about which traits determine yield nor which types of multispectral features may allow us to forecast yield potential in a variety testing trial in the early growth phase. Therefore, this study aims (1) to identify the best performing multispectral traits for yield prediction and classification in wheat (2) to investigate, if yield types can be classified in relatively early stages and finally (3) to investigate, how traits measured at different time points can be combined to predict yield more accurately.

## 2 Methods

### 2.1 Field trials

Field trials were conducted at the research station of the Technical University of Munich in Dürnast, Freising (48.40630° N, 11.69535° E) in the growing seasons of 2020/2021 and 2021/2022 further referred to as seasons 2021 and 2022. The soil at this location can be characterized by a homogeneous Cambisol with 20.8% clay, 61.5% silt and 16.6% sand. Precipitation during this period was 595 mm and 415 mm in the first and the second season, respectively. The average temperature was 7.2°C in the first and 8.0°C in the second season (**Supplementary Figure 1**). A lot of precipitation around flowering characterized season 2021 whereas the season 2022 suffered too little precipitation at the end of the tillering stage. Climate data was collected from a weather station (Station id 5404) operated by the Climate Data Center of the German Weather Service located a few hundred meters from the trials. The temperature was aggregated to phenologically meaningful growing degree-days (GDD) (**Equation 1**) (Bonhomme, 2000):


(1) $$GDD=\;\sum_{d=1}^{n}Tmean_{d}$$

(2) $$Tmean_{d}=\;\frac{\sum^{}\frac{maxT_{d,h}+minT_{d,h}}{2}-baseT}{24}$$

where *Tmean_d_* is the mean temperature for day d after sowing as determined by **Equation 2**, *maxT_d,h _* and *minT_d,h _* are hourly maximum and minimum temperatures for day d and *baseT* is the base temperature, which was set to 0°C.

A panel consisting of 19 diverse European winter wheat elite varieties (*Triticum aestivum*) in 2021 and 18 varieties in 2022 was grown in plots with a size of 10 m x 1.85 m. All varieties grown in 2022 were grown in 2021 as well (**Table 1**). The plots were arranged in a randomized complete block design with four replicates, resulting in 76 plots in 2021. In 2022, the 72 plots were part of a bigger trial, which was arranged as a randomized strip-plot design with four replicates as well. Orthophotos of the trials can be found in **Supplementary Figure 2** in the appendix. All plots used for this study were fertilized by applying 180 kg N ha^-1^ in three equal splits at BBCH 25, 32 and 65. Plant protection was carried out according to local practice. Sowing took place on the 10.11.2020 and the 20.10.2021 and all plots were harvested at full maturity on the 03.08.2021 and the 26.07.2022, respectively.

**Table 1:** Grain yield, yield group and phenology of the single varieties.

Variety	Grain Yield		Yield group		Stem Elongation		Booting		Heading		Flowering		Early Grain Filling		Late Grain Filling	
	2021	2022	2021	2022	2021	2022	2021	2022	2021	2022	2021	2022	2021	2022	2021	2022
Absalon	532.0 (32.8)	700.8 (47.6)			635.0 (27.7)	749.8 (7.2)	917.5 (37.3)	1061.8 (18.5)	1046.5 (23.6)	1172 (9.3)	1153.3 (38.7)	1273.3 (16.1)	1397.0 (59.2)	1404.5 (26.0)	1653.3 (79.2)	NA
Aurelius	541.9 (56.4)	759.7 (76.5)		H	609.3 (3.5)	762 (12)	963.5 (52.6)	1037.8 (75.0)	1069.3 (17.6)	1132.5 (51)	1109.0 (0.0)	1229.3 (34.2)	1368.8 (62.5)	1360.8 (26.9)	1673.3 (63.9)	NA
Axioma	473.0 (41.8)	631.0 (32.9)	L		607.5 (4.0)	765.3 (17)	950.3 (31.7)	1102.3 (59.7)	1042.8 (23.8)	1195.5 (66.9)	1121.5 (11.2)	1289.8 (80.2)	1350.0 (51.8)	1422.5 (71.8)	1636.5 (57.7)	NA
Bernstein	522.3 (33.7)	625.0 (102.2)			626.8 (16.6)	773.3 (17)	981.5 (6.9)	1145 (0.0)	1091.0 (30.2)	1225.8 (13.8)	1201.0 (70.6)	1311.5 (14.5)	1434.8 (27.3)	1460 (14.0)	1684.8 (93.6)	NA
Bologna	490.7 (33.3)	660.7 (29.0)			625.5 (16.7)	751.8 (32.4)	926.8 (61.1)	1081 (84.5)	1038.5 (30.0)	1178 (88.1)	1109.0 (0.0)	1271.8 (85.3)	1363.0 (69.7)	1408.3 (68.4)	1651.5 (64.4)	NA
CH-Nara	559.4 (30.2)	683.2 (108.0)			635.0 (27.7)	757.5 (9.9)	921.3 (16.7)	1126.5 (37.0)	1042.0 (16.2)	1208 (37.4)	1137.0 (30.9)	1283 (38.1)	1356.3 (47.8)	1405.8 (41.1)	1601.5 (23.7)	NA
Chevignon	598.5 (63.5)	672.5 (43.9)	H		615.5 (30.6)	776.5 (39.8)	911.8 (17.7)	1082 (44.3)	1050.8 (25.4)	1200 (46.2)	1144.8 (21.1)	1283.3 (45.3)	1358.3 (45.9)	1446 (26.8)	1579.3 (13.0)	NA
Costello	478.6 (38.8)	589.0 (52.8)		L	635.5 (22.3)	786.0 (32.0)	959.0 (31.2)	1145 (0.0)	1103.0 (38.0)	1256.5 (8.2)	1207.8 (67.8)	1338 (9.9)	1411.8 (39.7)	1477.7 (21.4)	1739.8 (2.5)	NA
Dagmar	617.1 (45.4)	725.2 (65.1)	H	H	611.0 (0.0)	803.3 (32.1)	969.0 (41.6)	1108.8 (82.1)	1052.0 (22.3)	1210.5 (77.2)	1114.0 (16.0)	1311 (67.5)	1345.3 (53.3)	1447.6 (81.6)	1653.5 (74.5)	NA
Elixer	540.4 (76.2)	721.3 (93.3)			643.0 (27.7)	768.5 (8.2)	953.0 (43.2)	1086.8 (75.7)	1118.3 (34.7)	1180.3 (60.5)	1219.5 (87.4)	1290.3 (57.4)	1444.0 (20.8)	1436.5 (61.0)	1742.0 (5.8)	NA
Hyvento	576.2 (51.1)	685.3 (128.0)			641.3 (21.0)	775.3 (29.3)	956.0 (34.8)	1126.5 (37.0)	1121.8 (6.0)	1220.3 (30.8)	1181.8 (41.7)	1299.3 (19.1)	1418.5 (23.7)	1438.8 (40.1)	1756.0 (119.6)	NA
Julie	544.2 (95.9)	661.8 (94.6)			635.0 (27.7)	754.0 (19.6)	915.3 (21.8)	1059 (67.1)	1057.3 (15.8)	1155 (58.7)	1142.8 (16.8)	1253.5 (59.1)	1347.3 (44.6)	1383.5 (39.0)	1648.5 (65.7)	NA
Julius	443.3 (14.6)	544.0 (25.5)	L	L	639.0 (17.3)	827.8 (45.9)	962.0 (26.9)	1157.8 (25.5)	1092.0 (40.1)	1277.3 (41.4)	1201.3 (65.4)	1366.8 (32.9)	1442.8 (28.5)	1475.6 (17.1)	1782.0 (62.2)	NA
Montalbano	584.1 (68.8)	681.1 (33.3)			612.8 (3.5)	768.8 (14.2)	954.0 (29.5)	1086.8 (75.7)	1104.0 (14.6)	1183.3 (55.7)	1192.0 (24.0)	1284 (43.9)	1428.0 (18.9)	1392.8 (21.5)	1720.5 (78.5)	NA
Mv Nador	552.4 (45.0)	591.9 (53.0)			643.0 (27.7)	751.0 (11.8)	962.3 (62.6)	1077.5 (80.4)	1084.3 (40.4)	1156.8 (59.8)	1157.3 (76.5)	1238.8 (43.8)	1343.8 (47.7)	1399.8 (53.7)	1624.5 (74.4)	NA
Nogal	504.2 (81.6)	557.8 (59.2)		L	633.3 (29.9)	751.0 (11.8)	972.8 (51.7)	1010 (27.7)	1058.8 (27.3)	1104.3 (19.1)	1084.0 (7.7)	1197.8 (12.7)	1281.5 (26.4)	1331.3 (13.4)	1602.0 (16.9)	NA
	2021	2022	2021	2022	2021	2022	2021	2022	2021	2022	2021	2022	2021	2022	2021	2022
RGT-Reform	411.4 (60.2)	671.6 (36.0)	L		629.0 (20.8)	792.3 (28.6)	941.0 (14.3)	1130 (39.9)	1105.0 (9.9)	1246.5 (28.1)	1179.0 (48.3)	1328.8 (24.1)	1426.8 (18.9)	1460 (14.0)	1641.3 (69.7)	NA
Rumor	543.9 (15.5)	NA			623.0 (24.0)	NA	926.8 (22.5)	NA	1045.3 (14.5)	NA	1124.8 (23.2)	NA	1403.8 (10.5)	NA	1635.5 (67.6)	NA
Skyfall	642.7 (49.6)	773.2 (47.1)	H	H	658.7 (82.6)	808.3 (66.8)	909.0 (41.2)	1133.5 (111.8)	1067.8 (34.2)	1222.8 (109.1)	1157.5 (32.8)	1323.5 (104.1)	1424.8 (21.0)	1438.7 (72.5)	1642.8 (84.7)	NA
All	535.5 (73.8)	663.1 (87.7)			628.5 (26.4)	773.4 (33)	945.1 (39.3)	1097.7 (65.9)	1073.2 (35.8)	1195.8 (64.1)	1154.6 (54.3)	1287.4 (55.3)	1386.6 (56.6)	1421.7 (55.3)	1666.8 (80.1)	NA

Values represent the mean of the four replicates; the values in parenthesis represent the standard deviation of the four replicates. Group “L” stands for low yielding, group “H” for high yielding; varieties without character have not been classified for the respective year.

### 2.2 Grain yield, phenology assessment and Leaf area index measurements

The entire plots were harvested using a combine harvester. The water content of the grains was determined by weighing the grains after harvest, drying them at 65°C until constant weight was reached and weighing them again. The final yield was normalized to a moisture content of 14%. In each year, the three varieties with the lowest average yield were classified as low yielding and the three varieties with the highest average yield as high yielding. The phenology of each plot was visually rated using the BBCH scale (Meier et al., 2009) on a plot level. Leaf area index (LAI) was measured using a Licor 2000 leaf area meter (LI-COR Biosciences, Lincoln, USA.) with a 45° view cap to minimize operator influence. One measurements was taken at the top of the canopy and four measurements were taken under the canopy at three different locations per plot, which were then averaged.

### 2.3 Multispectral image acquisition and processing

**Figure 1:**
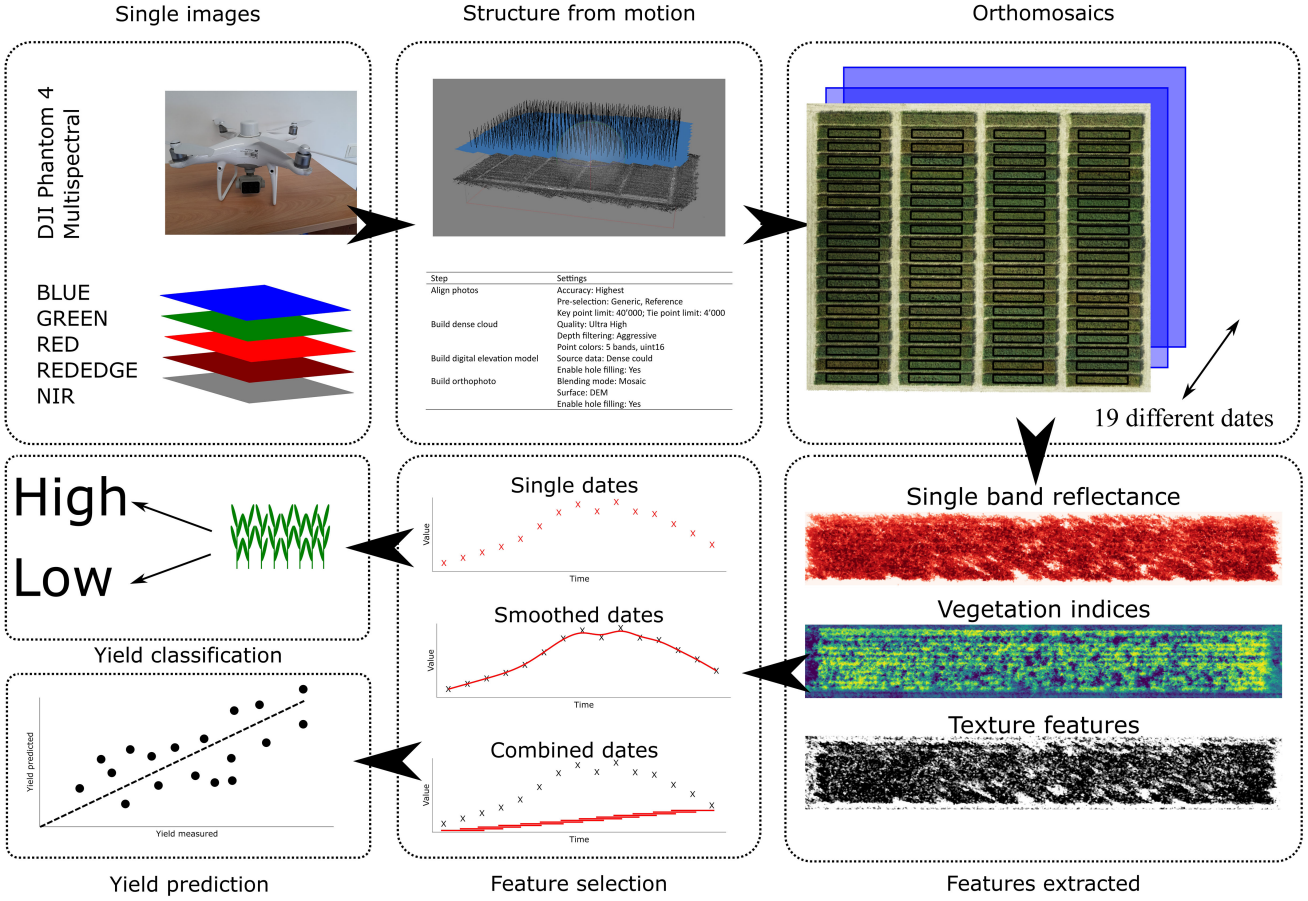
Workflow applied.

Spectral measurements were acquired using a Phantom 4 Multispectral RTK (DJI, Shenzhen, China) unmanned aerial vehicle (UAV) in the first year. The UAV captures reflectance in wavelengths of 450, 560, 650, 730 and 840 nm and measures the incoming sunlight by a sensor on top of the UAV. Flight height was set to 10 m above ground level (AGL) resulting in a ground sampling distance (GSD) of 0.7 cm. In the second year, images were acquired using a MicaSense Dual Camera Kit (AgEagle Aerial Systems Inc., Wichita, USA) capturing reflectance in wavelengths of 444, 560, 650, 717, 842 nm. The camera was mounted to a DJI Matrice M300 RTK UAV (DJI, Shenzhen, China). Flight height was set to 30 m AGL resulting in a GSD of 2.5 cm. In both years, overlap in both directions was set to 90%. Before and after each flight, images of a panel with a known reflectance were taken. Flights were carried out twice per week during heading and flowering stages and once per week at other stages. First flight was carried out on the 25.03.2021 and the 24.02.2022 and the last flight on the 20.07.2021 and the 27.07.2022 when the canopies were fully senescent. This resulted totally in 19 flights in 2021 and 22 flights in 2022. Images were taken around the solar noon and under sunny conditions, if possible. The images from each flight were mosaicked using the Agisoft Metashape Professional 1.8.4 (Agisoft, St. Petersburg, Russia) structure-from-motion software and were radiometrically calibrated using the reflectance panels on the ground and the incident light sensor on the UAV. The processing parameters used for all flight dates were similar (**Figure 1**). The point cloud was georeferenced using the real-time kinetic global positioning system (RTK-GPS) integrated into the UAS, with the RTK correction signal provided by SAPOS (Deutsche Landesvermessung, 2023) in 2021 and ground control points were used in 2022. Orthomosaics acquired in 2021 were resampled to the same GSD as in 2022 by the average method implemented in gdal (Gdal/Ogr Contributors, 2023). Reflectance of individual bands was extracted by calculating the median of a specific region of interest (ROI) representing a plot using a custom Python 3.7 script (Python Software Foundation, https://www.python.org/).

#### 2.3.1 Selection and calculation of spectral indices

To compare our approach across a range of vegetation indices (VIs), we classified them into five main groups based on their calculation method and selected a representative VI for each group. The five groups were differential-type, simple-ratio type, normalized differential type, three-band type, and combination of two spectral indices type (**Table 2**). We calculated the indices using a custom Python 3.7 script (Python Software Foundation, https://www.python.org/) and computed the median value for each index over the regions of interest (ROIs) corresponding to the plots.

**Table 2:** Vegetation indices (VIs) calculated.

Index type	Index	Formula	Reference
**Difference**	**DVI**	Nir−Red	(Shibayama et al., 1999)
**Ratio**	**RVI**	$\frac{\text{Nir}}{\text{Red}}$	(Shibayama et al., 1999)
**Normalized**	**NDRE**	$\frac{\text{Nir}-\text{Rededge}}{\text{Nir}+\text{Rededge}}$	(Barnes et al., 2000)
**Three Band**	**MCARI**	$\left(\left(\text{Rededge}-\text{Red}\right)-0.2*\;\left(\text{Rededge}-\text{Green}\right)\right)*\left(\frac{\text{Rededge}}{\text{Red}}\right)$	(Daughtry et al., 2000)
**Combination of indices**	**CCII**	$\frac{TCARI}{OSAVI}$	(Haboudane et al., 2002)
	TCARI	$3*\left[\left(\text{Rededge}-\text{Red}\right)-0.2*\left(\text{Rededge}-\text{Green}\right)*\left(\frac{\text{Rededge}}{\text{Red}}\right)\right]$	
	OSAVI	$\left(1+1.16\right)*(\frac{\left(\text{Nir}-\text{Red}\right)}{\text{Nir}+\text{Rededge}+0.16})$	

Green corresponds to 560 nm, the Red to 650 nm, Rededge to 730 and 717 and Nir to 840 and 842 nm wavelength in the first and the second year, respectively.

#### 2.3.2 Selection and calculation of texture features

Texture features (TFs) can be calculated on any data in a raster format, on single band reflectances and VIs likewise. In order to reduce the number of features to be tested, we focused on single band reflectances only. Hall-Beyer (2017) suggests to calculate TFs on the band showing the highest contrast and therefore we calculated the coefficient of variation (CV) for each plot and band. Over all dates, the RED band showed on average the highest CV in 2021 (0.324) and the second highest in 2022 (0.180) after BLUE (0.187). The CV of all bands and dates can be found in **Supplementary Figure 3** in the appendix. Furthermore, Zhang et al. (2021) found that TFs calculated on RED bands were correlated with LAI as well as leaf dry matter. Therefore, we chose the RED band as a base for the calculation of all TFs included in this study. A 5 x 5 kernel size was used to calculate the GLCM features over the entire raster. This small kernel size was chosen because wheat leaf sizes are relatively small compared to our GSD. A quantization level of 32 was used, with the lowest level corresponding to the first percentile of the respective raster and the highest level corresponding to the 99th percentile. This ensured that we could still capture the variation in the image. GLCMs were constructed with a moving distance of 1 pixel and moving directions of 0°, 45° and 90° to eliminate possible effects of direction. The CONTRAST, CORRELATION, DISSIMILARITY, ENERGY, and HOMOGENEITY features were extracted from each GLCM (Haralick et al., 1973) and saved as the center pixel in a raster. From these rasters, the final value per plot was extracted by averaging all values within the ROI. All calculations were performed using a custom Python 3.7 script (Python Software Foundation, https://www.python.org/). The extracted features are listed in **Table 3**.

**Table 3:** Calculation of grey correlation matrix features according to Haralick et al. (1973).

Texture feature calculated on RED raster	Formula	Explanation
Contrast	$\sum_{i,j=0}^{N-1}P_{ij}{\left(i-j\right)}^{2}$	Amount of local variation in pixel values
Correlation	$\sum_{i,j=0}^{N-1}P_{ij}\frac{\left(i-\;\mu \right)\left(j-\mu \right)}{\sigma^{2}}$	Linear dependency of grey level values in the GLCM
Dissimilarity	$\sum_{i,j=0}^{N-1}P_{i,j}\left|i-j\right|$	Local roughness of the pixel values
Energy	$\sum_{i,j=0}^{N-1}{\left(P_{ij}\right)}^{2}$	Local steadiness of the gray levels
Homogeneity	$\sum_{i,j=0}^{N-1}\frac{P_{ij}}{1+{\left(i-j\right)}^{2}}$	Homogeneity of the pixel values

#### 2.3.3 Temporal processing of the extracted features

Temporal feature selection was carried out in R (R Core Team, 2021). Three temporal feature selection strategies were evaluated (**Figure 1**). The first strategy involved selecting data from individual dates, resulting in one feature per observation. The second strategy involved smoothing the values per plot using splines, implemented in the package *statgenHTP* (Millet et al., 2022), with the default settings applied. Summed GDD from harvest were used as the time axis. Finally, features were selected using a moving time window with a width of 3. For each recorded date, the model included features from the current date and the previous as well as the following date, resulting in a total of three features per observation. This strategy is referred to as the moving window model.

### 2.4 Yield prediction model and yield potential classification model

To predict yield on a plot level and classify yield performance groups, we employed Random Forest (RF) machine learning models in R 4.2 (R Core Team, 2021). We optimized the number of trees per forest to 500 and used the R package *caret* (Kuhn, 2008). The number of trees per forest was set to 500 and the number of features per node was optimized by minimizing the root mean square error (RMSE) for the regression models and the accuracy for the classification models if more than one feature was available as in the moving window model.

### 2.5 Statistical analysis

Pearson correlation coefficient between yield and spectral features was calculated using measurements taken during tillering and flowering. At this date, most varieties were in the mid to end flowering and the correlation of VIs and yield was maximal for most VIs. The performances of the regression RF models were assessed by the coefficient of determination (R^2^) (**Equation 3**) as well as the RMSE (**Equation 4**) using a 10-fold cross validation that was repeated 3 times and averaged:


(3) $$R^{2}=\;\frac{\sum_{i=1}^{n}{\left(x_{i}-\;{\bar{x}}_{i}\right)}^{2}*\;{\left(y_{i}-\;{\bar{y}}_{i}\right)}^{2}}{\sum_{i=1}^{n}{\left(x_{i}-\;{\bar{x}}_{i}\right)}^{2}*\;\sum_{i=1}^{n}{\left(y_{i}-\;{\bar{y}}_{i}\right)}^{2}}$$

(4) $$RMSE=\;\sqrt{\frac{\sum_{i=1}^{n}{\left(x_{i}-\;y_{i}\right)}^{2}}{n}}$$

Where *x_i_* and *y_i_* represent the observed and the predicted yield, ${\bar{x}}_{i}$ and ${\bar{y}}_{i}$ represent the mean of the observed and the predicted yield, respectively. *n* represents the number of samples. The performances of the classification RF models were assessed by the accuracy (**Equation 5**) of the prediction using a 10-fold cross validation that was repeated 3 times and averaged:


(5) $$Accuracy=\;\frac{True\;positive+True\;negative}{Total\;number\;of\;classified\;objects}$$

## 3 Results

### 3.1 Yield, LAI and phenology

**Figure 2:**
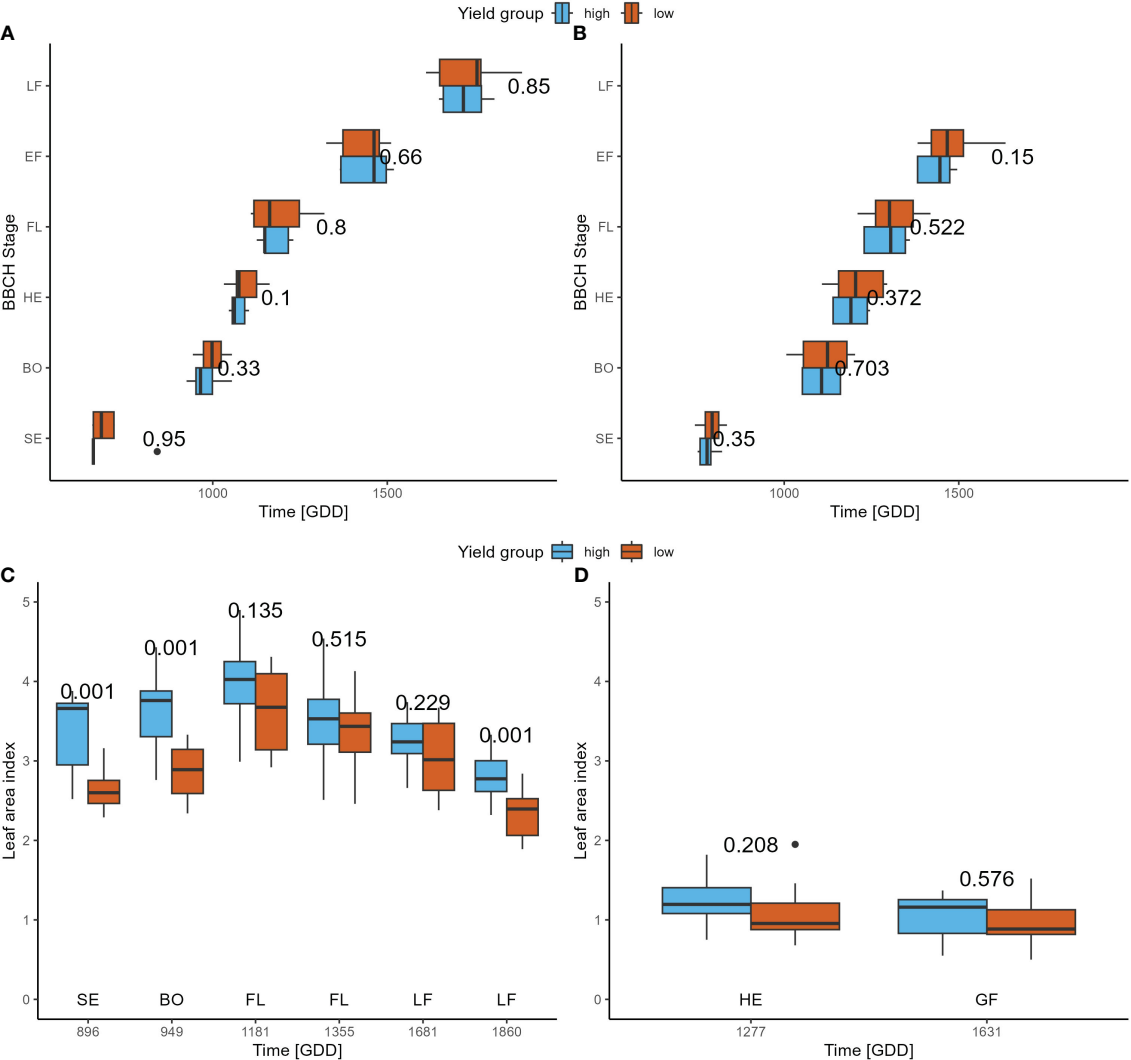
Subfigures **(A, B)** display the phenologies of the low and high yielding varieties during the two seasons of trial. Subfigures **(C, D)** display the LAI at different time points during the growth season. Numbers next or above the boxplot pairs show the p-value of a t-test.

Substantial grain yield variation was observed between experimental plots, with the season 2021 yielding about 128 g m^-2^ less than the second season (**Table 1**). Variety Julius was classified into the low yielding group in both years, while Skyfall and Dagmar were classified as high yielding in both years. The temperature sum to achieve a specific growth stage did not show significant differences between the yield groups. However, in both years a tendency towards an advanced phenology in the low yielding varieties could be observed (**Figure 2**). The Leaf Area Index (LAI) was notably higher in the first year of the trial compared to the second. In 2021, the high-yielding varieties showed a significantly higher LAI during the stem elongation, the booting and the late grain filling stage than the low-yielding varieties. This difference could not be observed in the second year (**Figure 2**).

### 3.2 Correlations between grain yield, the UAV-based reflectance, vegetation indices and texture features at tillering and flowering

**Figure 3:**
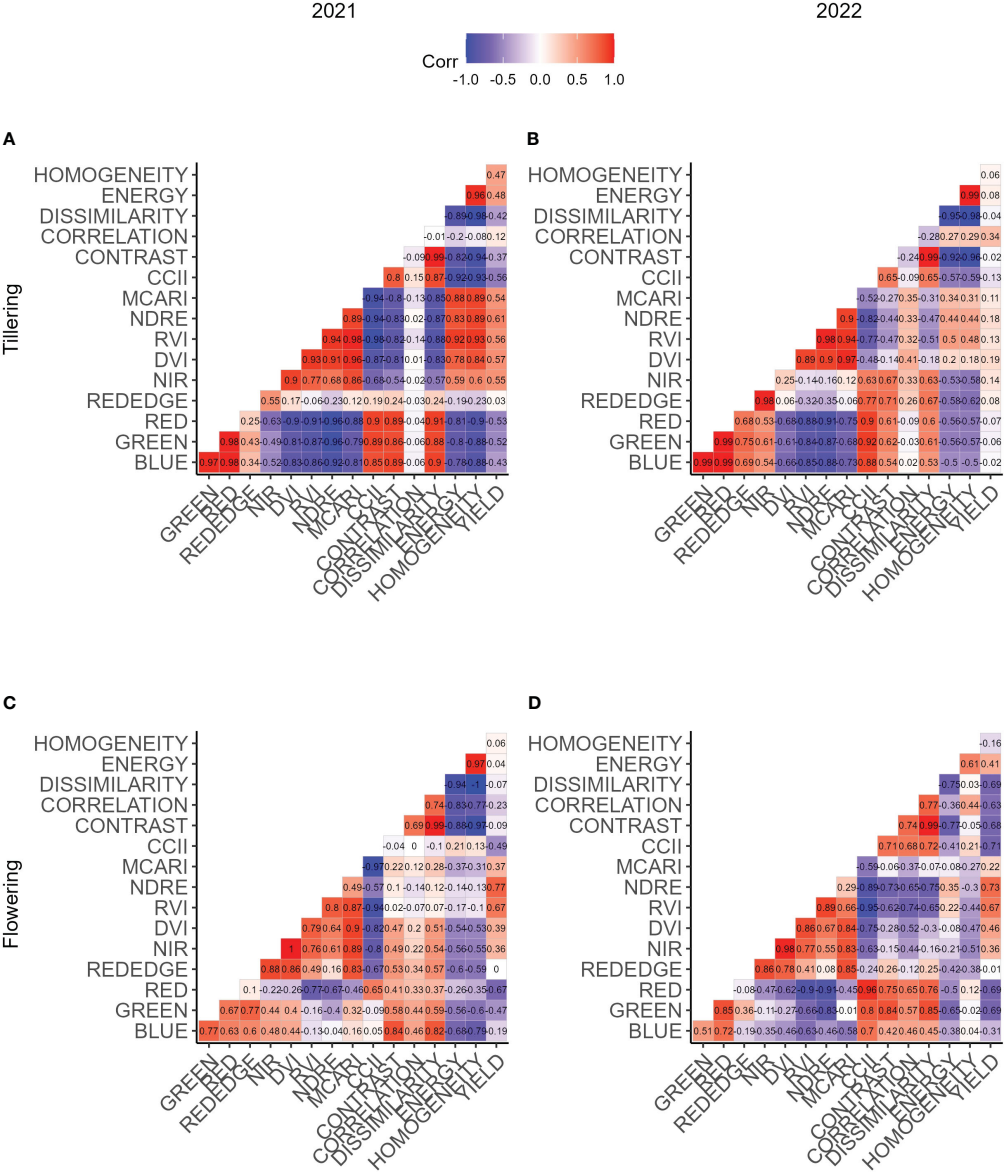
Correlation matrices of all features extracted for a single date and yield. Subfigures **(A, B)** display measurements that were taken at tillering, **(C, D)** measurements taken at flowering. Subfigures **(A, C)** belong to the first, **(B, D)** to the second year of trial.

**Figures 3A–D** show the Pearson correlation coefficient examining the relationship between reflectance, vegetation indices and TFs for the two years of trial at the end of the tillering and end of the flowering stages for the two years of trial. The analysis reveals that most features exhibit high correlations with one another during the tillering stage in both years, with few exceptions. Exceptions are the REDEDGE band in 2021, the REDEDGE and NIR bands in 2022 and the CORRELATION TF in both years. Correlation to yield at the tillering stage is high for all features in 2021 with a maximal R of 0.61 for the NIR band (**Figure 3A**). In 2022, correlations to yield are generally low, the highest correlation was found for the CORRELATION TF (R = 0.38) (**Figure 3B**).

At the flowering stage, correlation coefficients among features tend to decrease, especially between features of distinct feature groups. For instance, VIs are strongly correlated among themselves, while TFs similarly demonstrate robust correlations within their group. However, features belonging to the VI group are weakly with features belonging to the TF group. The absolute correlation of the features to yield generally decreases for TFs, increases for VIs and single band reflectances show few differences. In 2022, the absolute correlations increase towards the flowering stage, except for the REDEGE reflectance. For both years, the NDRE was the feature showing the highest correlation to yield, whereas the REDEDGE reflectance was not correlated to yield.

### 3.3 Time series of UAV-based reflectance, vegetative indices, texture features

**Figure 4:**
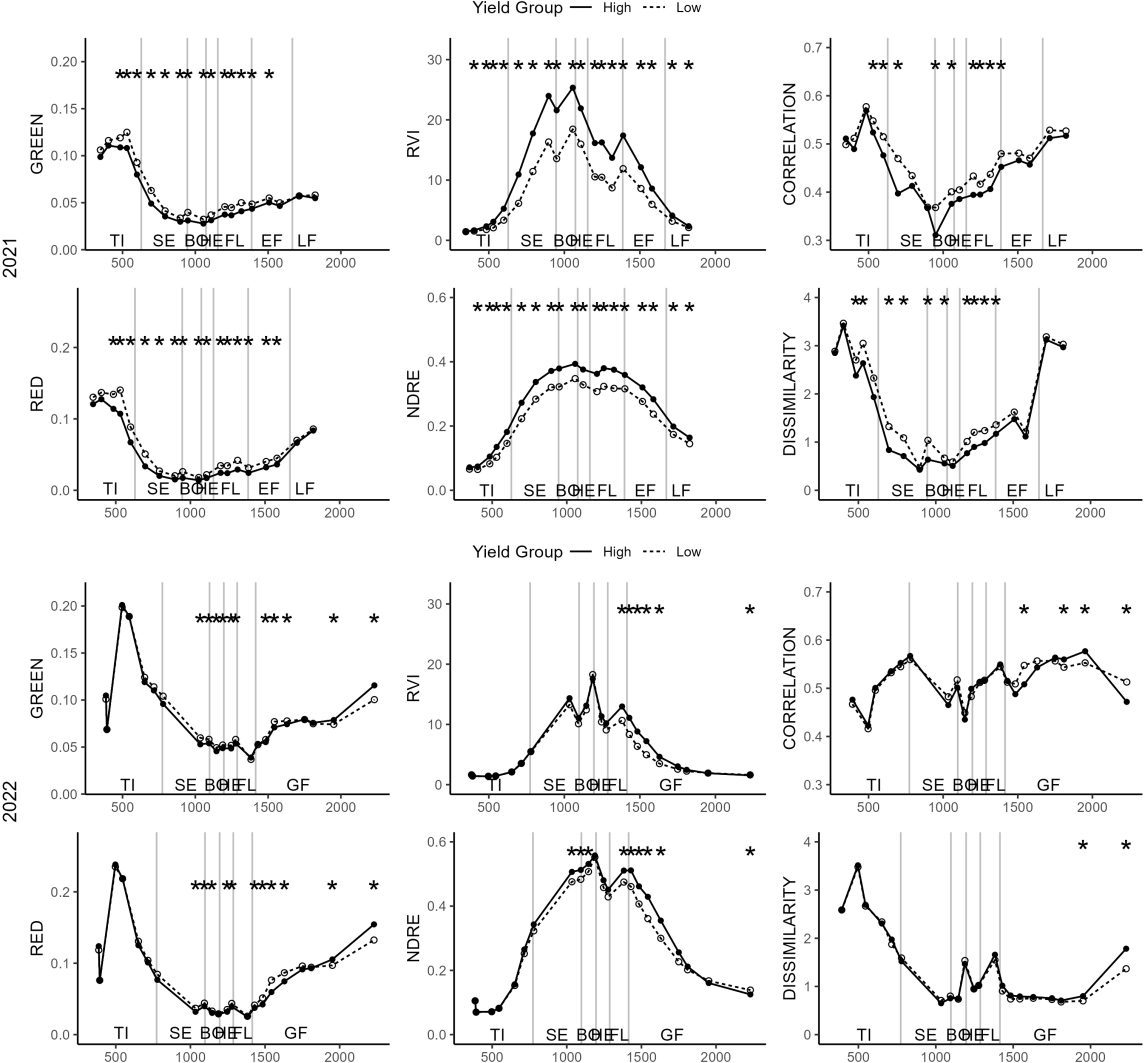
Dynamics of single band reflectances (left), vegetation indices (middle) and texture features (right) for different dates. The solid line shows the high yield group, the dashed line the mean value for the low yield group. The asterisks display significant differences after a t-test (p < 0.05) in the respective values and dates between the two yield groups. The plots are grouped into the first year of trial (top) and the second year of trial (bottom). Asterisks may overlap but only one significance level is given.

Reflectance of the GREEN and the RED band decreased with plant growth during the tillering and stem elongation stages followed by an increased with the onset of the grain filling stage. This trend resulted in a minimal reflectance around booting and flowering stages (**Figure 4**). In 2021, the high yielding group consistently displayed significantly lower GREEN and RED reflectances across various stages ranging from tillering to late grain filling. However, in the subsequent season, these marked differences between yield groups only emerged from the latter half of the stem elongation stage onward.

Both the RVI and NDRE indices showed significant disparities between the low and high yielding groups in 2021 at all recorded dates, except for the first one. In 2022, these distinctions were noticeable from the flowering stage to the initial segment of the stem elongation stage. Particularly, NDRE exhibited differences from the conclusion of the stem elongation stage onward (**Figure 4**). The TF CORRELATION decreased until booting and increased towards the end of grain filling in 2021.The feature does not show a clear development with time in 2022. The DISSIMILARITY TF decreases from the tillering until the booting stage and slight increases until harvest in both years. Significant differences between the two yield groups were found in 2021 for stages ranging from tillering to the end of flowering. In 2022, differences were found on few dates after flowering only (**Figure 4**).

### 3.4 RF regression model for yield prediction using individual flights and time series of UAV traits

**Figure 5:**
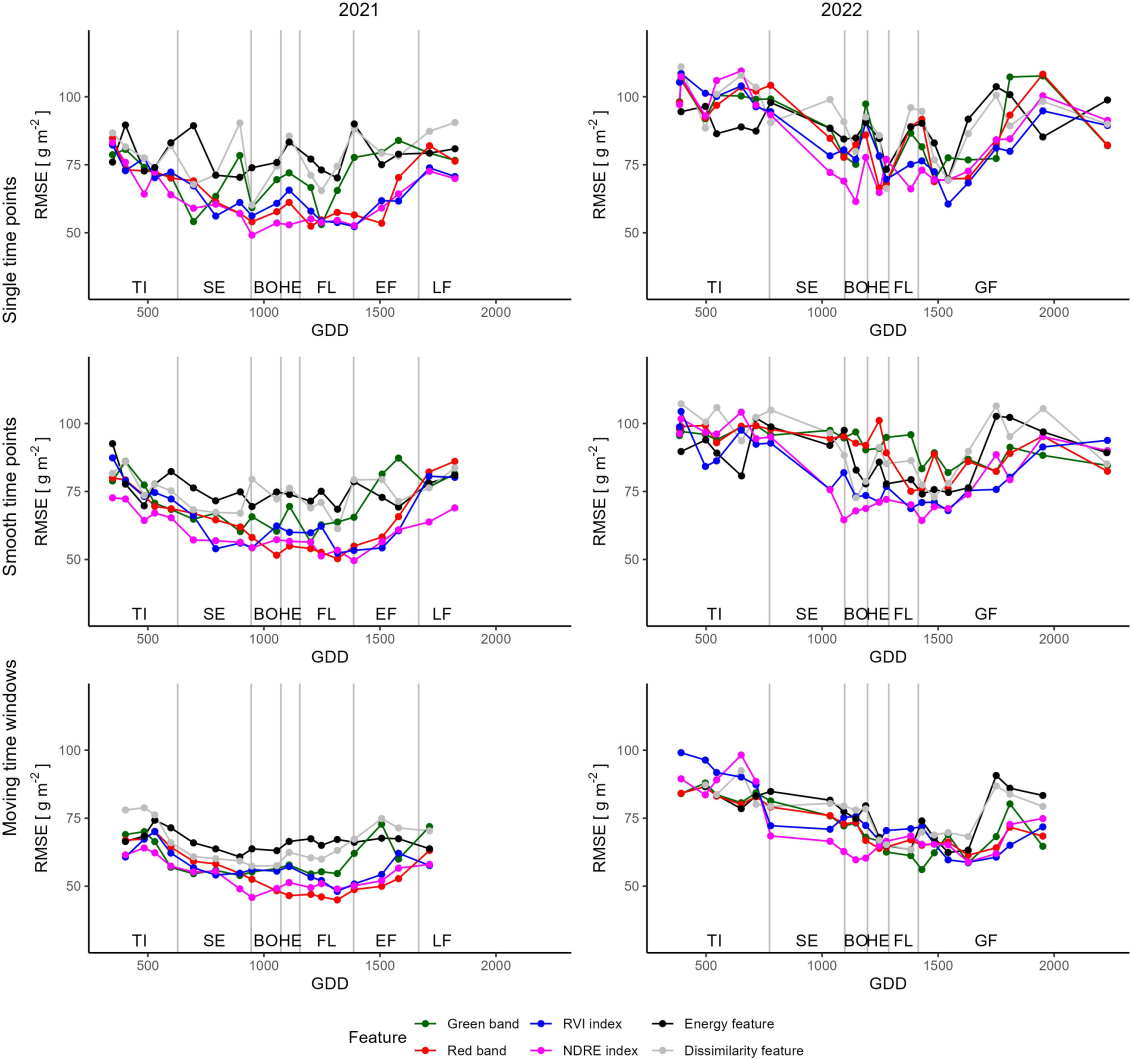
Root mean square errors of yield prediction models built using single dates (top), smoothed dates (middle) and combining adjacent dates (bottom). The figures on the right display models built for the first, the ones on the left for the second year of trial.

The performance of the yield prediction models depends highly on the features and the time point selected. Overall, the 2021 season demonstrated superior results, exhibiting lower average RMSE in contrast to the 2022 season. Generally, predictions improve from the tillering to the booting stage and deteriorate after flowering (**Figure 5**). In 2021, the most successful feature was the Normalized Difference Red Edge (NDRE) index at the booting stage, displaying an RMSE of 49.1 g m^-2^. In 2022, the Difference Vegetation Index (DVI) at the grain filling stage showed the best performance (RMSE = 60.6 g m^-2^). Individual bands, such as the RED band, produced good results, particularly in the 2021 season. However, on average they were outperformed by the VIs in both seasons (**Table 4**). The TFs were the feature group that led to regression models with the highest RMSE. On average, the RMSE was 10.3 g m^-2^ higher for the TFs than the VIs in 2021.

**Table 4:** Display of the time points yielding the lowest RMSE for yield prediction for all reflectance bands, vegetation indices and texture features.

Raster	Mean RMSE		Min RMSE		PS		VI	Mean RMSE		Min RMSE		PS		TF	Mean RMSE		Min RMSE		PS	
Single time points																				
	2021	2022	2021	2022	2021	2022		2021	2022	2021	2022	2021	2022		2021	2022	2021	2022	2021	2022
BLUE	76.8	95.2	58.7	80.4	BO	SE	DVI	70.6	86.7	61.6	64.4	SE	GF	CON	79.5	94.1	59.7	68.7	SE	HE
GREEN	71.3	88.5	53.0	68.0	FL	HE	RVI	64.7	85.6	52.3	**60.6**	EF	**GF**	COR	77.6	**92.1**	62.2	67.7	FL	TI
RED	**65.1**	**87.5**	**52.4**	66.5	**FL**	HE	NDRE	**61.8**	**83.5**	**49.1**	61.5	**BO**	BO	DIS	78.4	94.5	57.7	**66.3**	FL	**HE**
REDEDGE	77.6	87.6	56.0	**64.4**	LF	**SE**	MCARI	72.4	93.5	61.7	67.2	BO	GF	ENE	**76.4**	94.6	**55.5**	71.2	**SE**	GF
NIR	71.9	87.1	64.8	68.7	SE	GF	CCII	67.9	86.7	57.0	66.3	SE	GF	HOM	77.0	93.7	59.4	68.7	FL	FL
Mean	72.5	89.2	57.0	69.6				67.5	87.2	56.3	64.0				77.8	93.8	58.9	68.5		
Smoothed time points																				
	2021	2022	2021	2022	2021	2022		2021	2022	2021	2022	2021	2022		2021	2022	2021	2022	2021	2022
BLUE	76.6	96.3	67.1	86.4	SE	GF	DVI	69.3	84.7	63.7	65.9	EF	GF	CON	77.4	95.8	68.2	79.0	**SE**	BO
GREEN	70.7	92.0	56.4	82.0	FL	GF	RVI	65.4	**82.0**	52.3	68.0	**FL**	GF	COR	75.6	**87.6**	68.5	74.1	FL	GF
RED	**64.9**	90.9	**50.2**	75.1	**FL**	FL	NDRE	**60.1**	**82.0**	**49.6**	64.3	**EF**	GF	DIS	**74.5**	91.6	**61.3**	**72.7**	**SE**	**BO**
REDEDGE	78.1	93.9	65.8	83.8	SE	GF	MCARI	70.8	91.9	60.2	70.7	FL	GF	ENE	76.4	91.6	63.5	74.9	FL	GF
NIR	69.9	**90.8**	62.9	**72.7**	EF	**GF**	CCII	68.0	84.0	56.0	**61.4**	EF	**GF**	HOM	76.1	90.7	62.6	74.4	TI	BO
Mean	72.0	92.8	60.5	80.0				66.7	84.9	56.4	66.1				76.0	91.5	64.8	75.0		
Moving time window																				
	2021	2022	2021	2022	2021	2022		2021	2022	2021	2022	2021	2022		2021	2022	2021	2022	2021	2022
BLUE	65.1	83.0	52.7	76.5	BO	GF	DVI	62.8	76.6	58.1	62.7	EF	GF	CON	66.4	82.4	58.8	62.8	SE	FL
GREEN	60.4	**72.0**	53.9	**56.1**	SE	**GF**	RVI	57.2	74.6	48.1	58.7	FL	GF	COR	66.4	**80.6**	60.8	62.4	SE	GF
RED	**55.3**	72.1	**45.0**	61.4	**FL**	GF	NDRE	**54.0**	**71.5**	**45.8**	58.9	**BO**	GF	DIS	**66.1**	82.6	**57.4**	63.3	**BO**	FL
REDEDGE	66.1	74.5	57.5	61.1	SE	GF	MCARI	62.8	84.1	54.9	65.4	FL	GF	ENE	65.9	82.9	58.6	63.1	SE	GF
NIR	62.3	76.8	56.7	66.2	SE	GF	CCII	59.4	72.7	53.8	**50.8**	BO	**GF**	HOM	65.4	81.3	57.6	**56.9**	BO	**FL**
Mean	61.8	75.7	53.2	64.3				59.2	75.9	52.1	59.3				66.0	82.0	58.6	61.7		

Mean RMSE are all RMSE values averaged over the whole season. Min RMSE values show the lowest RMSE of a season. The phenology stages (PS) are reported at which the min RMSE was recorded.

Bold values indicate minimal Mean and Min RMSE values for a respective feature, featue selection and year combination. Phenology stages in bold indicate the stage when the minimal RMSE value was recorded.

Although data smoothing had a marginal influence the seasonal average RMSE, but worsened a few minimal RMSE. Additionally, smoothing altered the phenological stage at which the minimal RMSE was attained, illustrating shifts in the optimal time points for yield prediction. For instance, after smoothing, the NDRE feature in 2021 displayed an optimal time point for yield prediction during the early grain filling stage as opposed to the unsmoothed time points, which indicated the booting stage as optimal (**Table 4**).

Combining features from three adjacent time points notably enhanced the average RMSE models by approximately 10 g m^-2^ and reduced the minimal RMSE by 5 g m^-2^ for both years across all feature groups. Employing the moving window method revealed that the lowest RMSE of 45.0 g m^-2^ was achieved using the RED reflectance band at the flowering stage, while in 2022, the Vegetation Index (VI) CCII reached an RMSE of 50.8 g m^-2^. The combination of TFs from various dates yielded models that were comparable to models constructed with a single reflectance band or VI. Specifically, the DISSIMILARITY feature reached a minimal RMSE of 57.4 g m^-2^ at the booting stage in 2021, and the HOMOGENEITY feature achieved an RMSE of 56.9 g m^-2^ at the flowering stage in 2022 (**Table 4**).

### 3.5 RF classification model for classifying the high and low yielding varieties using individual flights and time series of UAV traits

The efficacy of classification models depends on the chosen features and time points, paralleling the observation in regression models. On average, the models in the first season exhibited higher accuracy compared to those in the subsequent season. However, the maximum accuracies achieved in both years were similar. Notably, in 2021, an accuracy of 0.938 was attained using the BLUE band during the late grain filling stage, while the HOMOGENEITY TF in 2022 achieved the maximum accuracy of 0.915 during the grain filling stage. In 2021, VIs generally outperformed single-band reflectances and TFs for yield predictions, whereas in 2022, TFs slightly surpassed the other two groups. The model performances in both years exhibited substantial variability across different dates. Although data smoothing mitigated some fluctuations, significant differences between adjacent dates persisted. Similar to the regression models, the average and maximum performance of the models remained largely consistent after the smoothing of data points.

**Figure 6:**
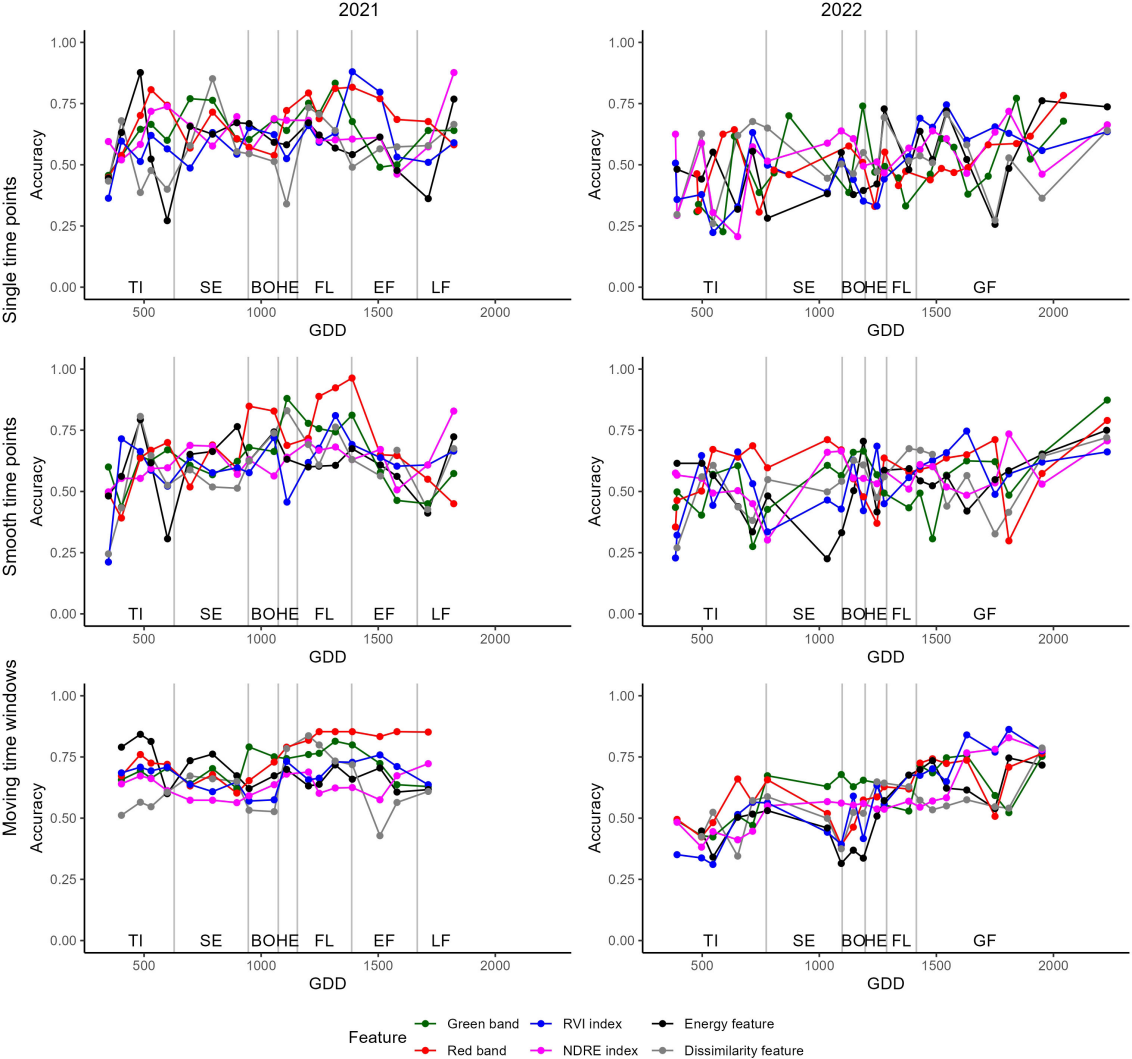
Accuracy of variety classification models built using single dates (top), smoothed dates (middle) and combining adjacent dates (bottom). The figures on the right display models built for the first, the ones on the left for the second year of trial.

Incorporating more than one date into the models generally yielded a slightly higher average accuracy for all features in both years. On average, the best-performing feature was the RED band with an accuracy of 0.758 in 2021 and the Modified Chlorophyll Absorption Ratio Index (MCARI) in 2022, displaying an average accuracy of 0.615. However, the maximal accuracies remained largely unaltered compared to those achieved using single dates (**Table 5**). Despite this, fluctuations between individual dates were reduced (**Figure 6**). A trend towards increased accuracy with time was evident in 2021, particularly notable with the RED band, consistently yielding high accuracies after heading. In the second year, accuracies improved and were higher than 0.5 for all features at the end of the heading stage. Moreover, fluctuations between dates were notably reduced in this subsequent year (**Figure 6**).

**Table 5:** Display of the time points yielding the lowest RMSE for yield prediction for all reflectance bands, vegetation indices and texture features.

Raster	Mean Accuracy		Max Accuracy		PS		VI	Mean Accuracy		Max Accuracy		PS		TF	Mean Accuracy		Max Accuracy		PS	
Single time points																				
	2021	2022	2021	2022	2021	2022		2021	2022	2021	2022	2021	2022		2021	2022	2021	2022	2021	2022
BLUE	0.609	0.501	**0.938**	0.693	**LF**	GF	DVI	0.640	0.502	0.825	**0.838**	EF	**GF**	CON	**0.594**	0.526	0.800	0.832	SE	GF
GREEN	0.643	0.488	0.833	0.772	FL	GF	RVI	0.595	0.504	0.880	0.745	EF	GF	COR	0.588	0.516	**0.877**	0.835	**TI**	HE
RED	**0.673**	0.506	0.817	**0.783**	EF	**GF**	NDRE	0.632	0.533	0.877	0.718	LF	GF	DIS	0.564	**0.538**	0.852	0.778	SE	GF
REDEDGE	0.584	**0.545**	0.798	0.707	LF	TI	MCARI	0.637	**0.567**	0.823	0.795	EF	GF	ENE	0.533	0.527	0.790	0.787	FL	SE
NIR	0.620	0.493	0.912	0.768	TI	BO	CCII	**0.673**	0.491	**0.885**	0.735	**FL**	GF	HOM	0.575	0.532	0.867	**0.915**	FL	**GF**
Mean	0.625	0.507	0.860	0.745				0.635	0.519	0.858	0.766				0.571	0.528	0.837	0.829		
Smoothed time points																				
	2021	2022	2021	2022	2021	2022		2021	2022	2021	2022	2021	2022		2021	2022	2021	2022	2021	2022
BLUE	0.674	0.551	0.853	0.813	LF	GF	DVI	0.600	0.521	0.815	0.860	TI	GF	CON	0.546	0.463	**0.875**	0.750	**EF**	GF
GREEN	0.640	0.538	0.880	**0.873**	HE	**GF**	RVI	0.609	0.535	0.810	0.747	FL	GF	COR	**0.612**	0.524	0.793	0.750	TI	GF
RED	**0.676**	**0.580**	**0.963**	0.790	**EF**	GF	NDRE	0.625	0.556	0.828	0.735	LF	GF	DIS	0.605	0.534	0.830	0.720	HE	GF
REDEDGE	0.594	0.553	0.830	0.813	HE	GF	MCARI	**0.647**	**0.586**	0.755	**0.868**	FL	**GF**	ENE	0.547	0.506	0.830	**0.840**	TI	**GF**
NIR	0.573	0.512	0.802	0.832	TI	GF	CCII	0.630	0.512	**0.858**	0.835	**FL**	GF	HOM	0.601	**0.537**	0.782	**0.840**	FL	**BO**
Mean	0.6314	0.547	0.866	0.824				0.622	0.542	0.813	0.809				0.582	0.513	0.822	0.780		
Moving time window																				
	2021	2022	2021	2022	2021	2022		2021	2022	2021	2022	2021	2022		2021	2022	2021	2022	2021	2022
BLUE	0.697	0.535	**0.884**	0.712	**LF**	GF	DVI	0.684	0.499	**0.833**	0.815	**TI**	GF	CON	0.617	0.572	0.903	0.813	SE	GF
GREEN	0.712	**0.604**	0.814	0.757	FL	GF	RVI	0.674	**0.581**	0.758	0.863	EF	GF	COR	**0.652**	0.567	0.867	0.833	TI	GF
RED	**0.758**	0.598	0.853	0.764	FL	GF	NDRE	0.630	0.573	0.722	0.828	LF	GF	DIS	0.602	**0.584**	0.840	0.793	EF	GF
REDEDGE	0.659	0.577	0.800	0.777	HE	GF	MCARI	**0.719**	0.615	0.795	0.783	TI	GF	ENE	0.634	0.558	**0.918**	**0.852**	**SE**	**GF**
NIR	0.668	0.578	0.788	**0.868**	TI	**GF**	CCII	0.708	0.559	0.827	**0.900**	HE	**GF**	HOM	0.638	0.553	0.882	0.815	SE	GF
Mean	0.698	0.578	0.828	0.776				0.683	0.565	0.787	0.838				0.629	0.567	0.882	0.821		

Mean RMSE are all RMSE values averaged over the whole season. Min RMSE values show the lowest RMSE of a season. The phenology stages (PS) are reported at which the min RMSE was recorded.

Bold values indicate maximal Mean and Max Accuracy values for a respective feature, featue selection and year combination. Phenology stages in bold indicate the stage when the Maximal RMSE value was recorded.

## 4 Discussion

### 4.1 Dynamic responses of individual bands

Rededge bands have been widely studied for assessing crop performance and yield in various crops, including wheat (Horler et al., 1983; Pavuluri et al., 2015). Canopy reflectance in the red, rededge and near-infrared (NIR) wavelength range is influenced by two primary optical properties of canopies: chlorophyll absorption in the red region and multiple scattering effects on the NIR due to canopy structural properties. The red-edge region is more sensitive to chlorophyll content than to leaf area (Xie et al., 2018). Hence, the variability in LAI might have been bigger or more important for yield formation compared to the chlorophyll content in our specific panel. Moreover, the correlation of reflectance in the red-edge region with yield is known to change quickly with the exact wavelength measured (Pavuluri et al., 2015), making the selection of the exact wavelength difficult and leading to inconsistent results.

In contrast, visible bands (Blue, Green and Red) can be more sensitive to yield-related variations in chlorophyll, and biomass accumulation during the tillering and the stem elongation stage until the beginning of the booting stage. They are known to be correlated to a certain extent to both, chlorophyll concentration and LAI (Daughtry et al., 2000). Accordingly, our results showed that the RED, GREEN, and BLUE bands were among the most effective spectral features for yield prediction, exhibiting significant differences between high- and low-yielding varieties at almost all measurement dates in the first year of trial and during several in the second year. Their reflectances decrease during the transition from the stem elongation to the beginning of the booting stage when LAI and chlorophyll density is known to be maximal (Hinzman et al., 1984; Hinzman et al., 1986) due to the optical properties of chlorophyll. From heading until harvest the reflectances in these bands increase due to senescence when chlorophyll degradation takes place (Spano et al., 2003).

The NIR region is known to be sensitive to leaf area and especially ground cover (Korobov and Railyan, 1993), making it a useful band for predicting biomass and therefore yield. Our results indicate that the NIR band performed best during the stem elongation stage for yield prediction and at the booting stage when there were significant differences in (LAI) between the two yield groups. In the second season, the differences in the reflectances were significantly different only once before booting in the second season possibly due to a lack of differences in LAI between the two yield groups. This aligns with the findings by Korobov and Railyan (1993), who reported a higher correlation of NIR reflectance with dry matter and ground cover the during booting stage compared to later stages. Thus, normalizing the difference of the NIR and the REDEDGE reflectance in the form of the NDRE index, showed a good performance for chlorophyll estimation (Barnes et al., 2000).

Usually, VIs containing information from the rededge region of the spectrum are considered being more sensitive to chlorophyll absorption in dense canopies (Nguy-Robertson et al., 2012). It is expected that combining the highly LAI-sensitive NIR band with the rededge band that contains more information about leaf pigments in the canopy and therefore improves the performance of our yield prediction model at the flowering to early grain filling stages.

### 4.2 The influence of growth stages on yield prediction and classification

The performance of yield prediction and classification depends highly on the phenological stage of the crop. Our study found that the flowering stage and early grain filling stage allowed for the best predictions of yield and classification of varieties in winter wheat, which is consistent with the findings of several other studies (Hassan et al., 2019; Prey et al., 2020; Prey et al., 2022). From a physiological stand point of view, at the time around flowering the crop has to provide enough assimilates in order to maximize the number of fertile florets per spike, leading to a higher number of kernels per spike and finally a higher yield (Fischer, 1985). Therefore, estimating biomass and chlorophyll content at these stages is optimal for yield estimation. Unfortunately, the spectral signal often saturates at these stages making the estimation challenging, especially in high yielding years (Prey et al., 2020).

Early differences in biomass and LAI dynamics between wheat genotypes are well-documented (Pang et al., 2014; Grieder et al., 2015) and Raun et al. (2001) proposed to follow the biomass formation after dormancy for yield prediction. Marti et al. (2007) hypothesized that a high biomass at the end of the tillering stage detected by NDVI estimates the growth during the stem elongation stage which in turn is crucial for the number of kernels per area produced (González et al., 2005). Yield prediction at tillering therefore already yielded some success especially in the first year of the trial. From tillering to harvest, wheat is known to compensate for, as an example, a low stand count by altering the number of yield components (Holen et al., 2001), therefore predictions at these early stages are prone to changes later in the season, subsequently leading to prediction errors.

After flowering, the RMSE of the models for prediction increases again in the first year whereas they start to increase later during grain filling in the second year. The flowering stage is longer in the first compared to the second year of trial (232 GDD and 134.4 GDD). The reason for this difference is unclear, the end of flowering is generally difficult to rate, since the dry anthers tend to fall to the ground or are washed of by rain. Therefore, the end of flowering might have been later in the second year as well. Differences in stay-green characteristics can influence yield, especially by influencing the weight of single grains (Wu et al., 2012) which were higher in the high yielding varieties compare to the lower yielding ones. A further indication of this difference is the slightly advanced phenology of the lower yielding varieties in both years and the higher LAI measured in the first year of the trial.

The classification models showed a less clear trend during the season and fluctuations between dates are more severe than for the regression models. Garriga et al. (2017) did not find a difference in classification model performance between the anthesis and the grain filling stages. Successful classification in our case can be achieved directly by identifying high yielding plots or indirectly by identifying varieties. The algorithm learns either to recognize high yield or to classify a certain variety, regardless of their yield potential. Wheat variety classification for yield classification for breeding purposes other than by Garriga et al. (2017) is scarce. Works often focus on the classification of kernels (Porker et al., 2017; Khojastehnazhand and Roostaei, 2022), which is done under laboratory environments and therefore less prone to errors induced by the measurement conditions. Still, if the optimal feature at the optimal time point is selected, classification of low and high yielding varieties can be a promising tool for plant breeding applications (Garriga et al., 2017).

### 4.3 Comparison of variable- and feature types for yield prediction and classification

Our study found that single-band reflectance, such as the RED band, was as effective as or even more effective than vegetation indices (VIs) for predicting yield, especially in the first year of trial. The RED band is known to be related to leaf area index (LAI), although this relationship is often non-linear (Hinzman et al., 1984) and therefore requires non-linear methods such as RF to perform well for yield prediction. Pavuluri et al. (2015) found a saturation of RED reflectance when predicting yield, which can also be found in our prediction models. In contrast, VIs typically show good linear correlations with grain yield, with NDVI being widely used for yield prediction (Duan et al., 2017; Hassan et al., 2019). Furthermore, the VIs show a more consistent performance between dates compared to the single band reflectances. The NDRE was the best performing feature for yield assessment, which is in accordance with other studies (Prey et al., 2022) possibly due to its strong correlation to biomass (Argento et al., 2021). Many VIs have been screened by Prey et al. (2020) and few have been showing a consistent performance over the years, which makes a general selection difficult, similar to our study. Further, VIs narrow down the information that is accessible and Vatter et al. (2022) found good performances for yield prediction when using 11 wavebands from a multispectral camera that were fed to a deep learning model. This might be good strategy to obtain the optimal prediction model from multispectral cameras without any prior knowledge and the need for feature selection.

TFs are complex in their calculation and they offer a variety of possible ways of calculation, possible combinations with underlying rasters and ways to be calculated. Detailed information on how TFs are calculated is often lacking (Zheng et al., 2019; Wang et al., 2021; Zhang et al., 2021). Therefore, TFs still have to be examined in detail and their parameters optimized under different experimental conditions and scenarios of sensing data collection. We calculated TFs in a standardized way, but still found a high variability between dates. They are further known to be highly dependent on the GSD and therefore, the flight height (Zheng et al., 2019). In our study smoothing aided in enhancing the stability of the yield prediction models, although it did not improve their performances. A novel approach was presented by Herrero-Huerta et al. (2020) who calculated so-called canopy roughness directly on the point cloud from the structure from motion processing and showed its correlation to biomass. Often, the TFs are difficult to interpret and their link to yield relevant canopy traits is often unclear. Originally, the TFs were developed for classification (Haralick et al., 1973) and therefore classifying varieties corresponds more to their intended purpose than yield prediction. TFs are further often used in combination because there might be additional information (Wang et al., 2021; Liu et al., 2022), especially in the later stage, when they are not strongly correlated to single band reflectances and VIs anymore, as indicated by our results.

### 4.4 Effects of temporal feature selection for yield prediction and classification

Models using individual dates showed generally a worse performance than models containing three adjacent dates. Furthermore, the performance between different dates is fluctuating strongly, even if the phenological stage is similar. Prey et al. (2022) found combining data from multiple dates to yield more improved predictions especially if the features used were performing poorly. In our case, the improvement for the yield prediction was in a similar range, regardless of the initial performance of the feature. In rice, Zhou et al. (2017) found that combining data from different growth stages by a multilinear regression model, can improve the estimation accuracy. For practical applications, finding the optimal date might be difficult and requires very close monitoring of the phenology, which can be very diverse among varieties as in our variety-testing panel. Therefore combining multiple dates might be especially performant, if features are used that show a high fluctuation between dates such as the TFs. The RF algorithm however is capable of dealing with different suitability of dates and therefore neglect the ones that do not perform well by attributing different importance to the features. The downside of the method is that, obtaining the additional measurements requires substantial work. Therefore, the number and time points measured should be considered carefully and be optimized in future studies.

### 4.5 Limitations and outlook

The red-edge position and its shape is often used to estimate the stress status of field crops (Guyot et al., 1988; Boochs et al., 1990). However, it is obvious that the dynamics (time series) of the Red-edge band is difficult to interpret compared to the visible bands. During the early stages of tillering, the red-edge reflectance increased, possibly due to an increase of ground cover, whereas later it decreased again, when the canopy height increased during the SE stage. At the beginning of the heading stage, another increase in the red-edge reflectance could be observed, accompanied with the increase of reflectance in the visible bands. However, in contrast to other bands, the Red-edge reflectance decreases with the onset of senescence at the early grain-filling stage, possibly due to a reduction in chlorophyll and a shrinking canopy structure (Wang et al., 2022). However, fluctuation also occurs during the mentioned stable period running from the beginning of booting to the end of flowering. These fluctuations can be of various origins. For instance, the appearance of the canopy might change significantly due to the emergence of the spikes. Although this study was unable to exploit the entire shape of the red-edge reflectance, due to limitations in our multispectral camera having one band in the red-edge region, future work should further advance the understanding of the dynamics of red-edge reflectance and responsible canopy characteristics. Also, features should, in addition to their performance for yield prediction be assessed regarding their heritability (H^2^) since breeders are interested in knowing the genetic variation underlying a trait or in our case a spectral feature. Generally, this study shows that a trait time series followed by smoothing and a moving window allows for more stable predictions when also not better predictions.

## 5 Conclusions

Most spectral and TFs derived from the canopy multispectral images were related to variations in yield and delivered the best predictions of yield between booting and the beginning of senescence. Still, predictions before and after this stages yielded respectable results as well. Vegetation indices (VIs) generally outperformed single indices in assessing yield and in classifying varieties. Particularly the Normalized Difference Red Edge (NDRE) index performed well in both years and at several phenology stages. Single bands, especially the RED band showed a comparable performance but with more fluctuations between dates. In contrast, the REDEDGE reflectance showed poorer performance in yield and variety classification. TFs generally performed poorly for yield prediction, and their performances were inconsistent across dates in this study. TFs showed a good performance when classifying varieties. Further research is still needed to better understand the applicability of different TFs for yield- and traits predictions. Smoothing or combining data across a time series can enhance the performance of yield prediction and classification models, particularly in the early growth stages. Future studies should combine different feature types to leverage complementary information captured by different types of multispectral features and variables.

## Data availability statement

The raw data supporting the conclusions of this article will be made available by the authors, without undue reservation.

## Author contributions

KY designed the experiment. MC managed the UAV flights for aerial imagery, analyzed the data, and conducted the ground-based field measurements with supervision from KY. MC and KY wrote and revised the manuscript. All authors contributed to the article and approved the submitted version.
