# Supplementary figures and images for: Multi temporal multispectral UAV remote sensing allows for yield assessment across European wheat varieties already before flowering
Source: Front Plant Sci. 2024 Jan 3;14:1214931. doi: 10.3389/fpls.2023.1214931 (PMC10791776; doi:10.3389/fpls.2023.1214931)

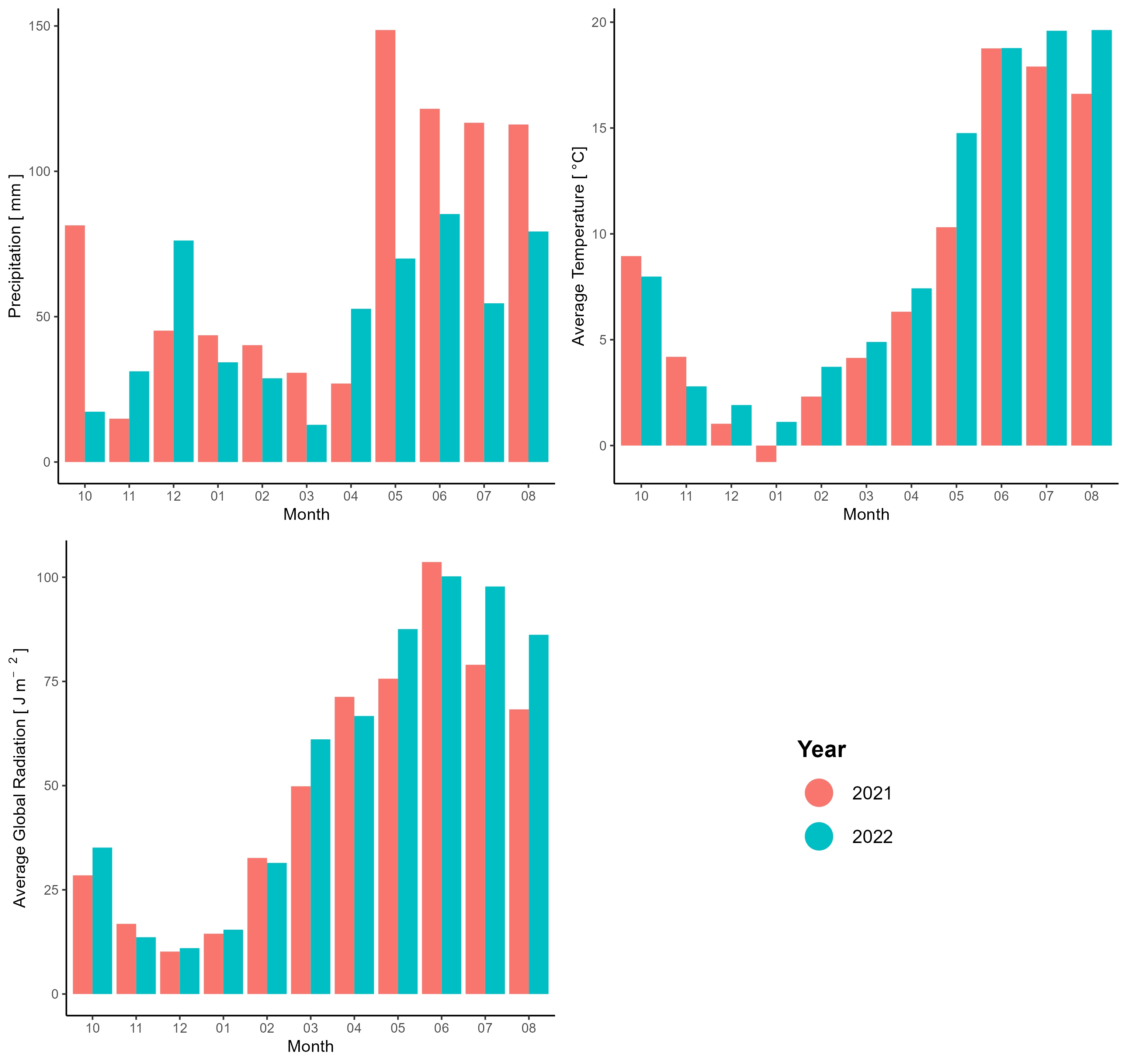

Supplement: Supplementary file 1 [file Image_1.jpeg]

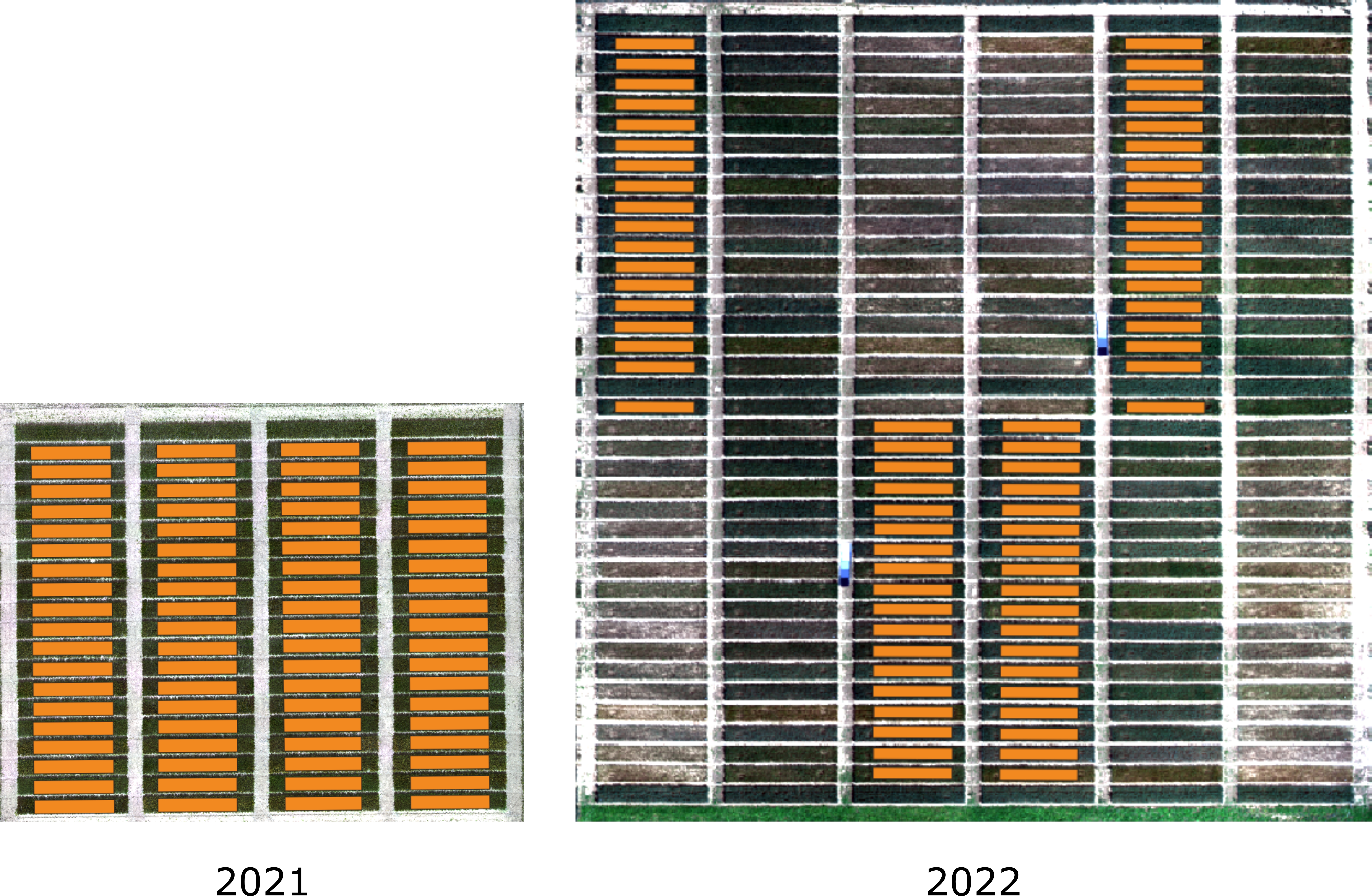

Supplement: Supplementary file 2 [file Image_2.jpg]

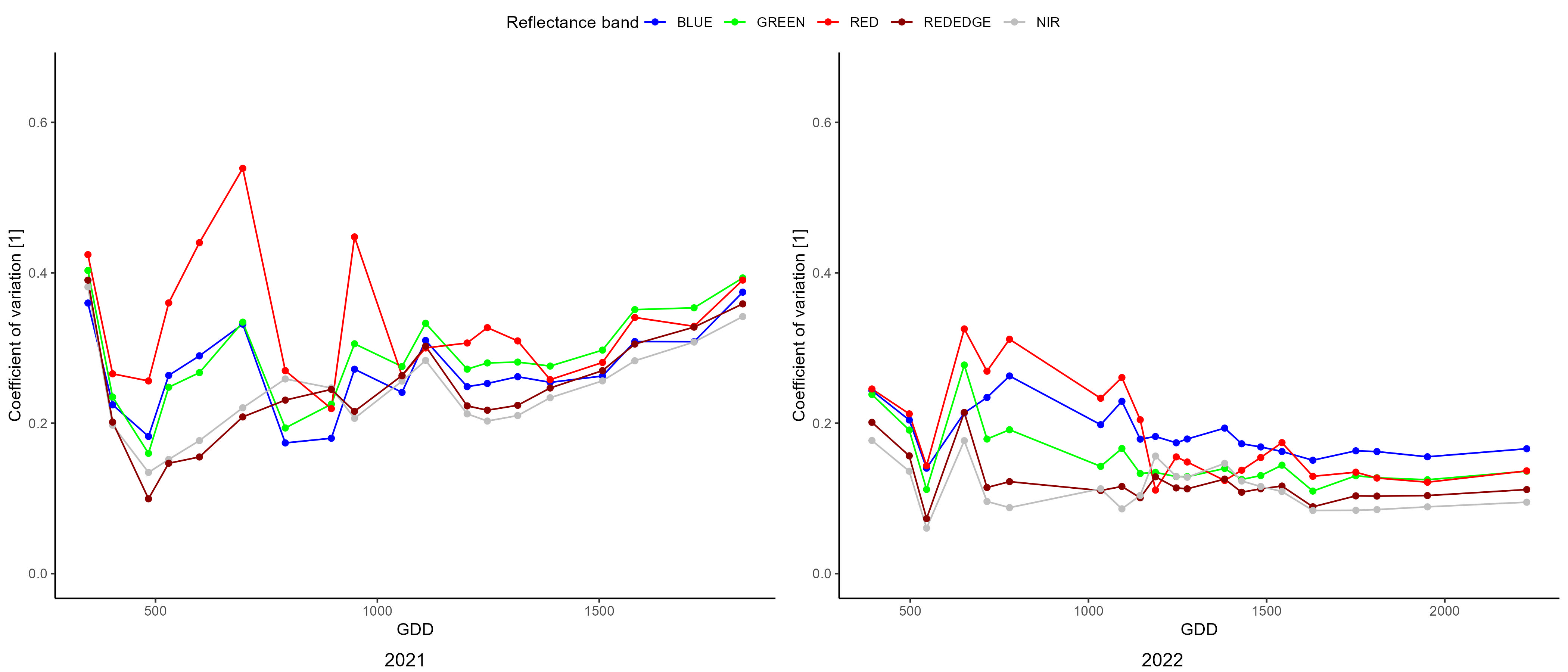

Supplement: Supplementary file 3 [file Image_3.jpeg]
